# 1-Deoxysphingolipids Encountered Exogenously and Made *de Novo*: Dangerous Mysteries inside an Enigma*

**DOI:** 10.1074/jbc.R115.658823

**Published:** 2015-05-06

**Authors:** Jingjing Duan, Alfred H. Merrill

**Affiliations:** From the Schools of Biology and Chemistry & Biochemistry, and the Parker H. Petit Institute for Bioengineering and Bioscience, Georgia Institute of Technology, Atlanta, Georgia 30332

**Keywords:** cancer, ceramide, diabetes, serine, serine palmitoyltransferase, sphingolipid, 1-deoxysphingolipid, alanine, neuropathy, fumonisin

## Abstract

The traditional backbones of mammalian sphingolipids are 2-amino, 1,3-diols made by serine palmitoyltransferase (SPT). Many organisms additionally produce non-traditional, cytotoxic 1-deoxysphingoid bases and, surprisingly, mammalian SPT biosynthesizes some of them, too (*e.g.* 1-deoxysphinganine from l-alanine). These are rapidly *N*-acylated to 1-deoxy-“ceramides” with very uncommon biophysical properties. The functions of 1-deoxysphingolipids are not known, but they are certainly dangerous as contributors to sensory and autonomic neuropathies when elevated by inherited SPT mutations, and they are noticeable in diabetes, non-alcoholic steatohepatitis, serine deficiencies, and other diseases. As components of food as well as endogenously produced, these substances are mysteries within an enigma.

## Introduction

**FIGURE 1.**
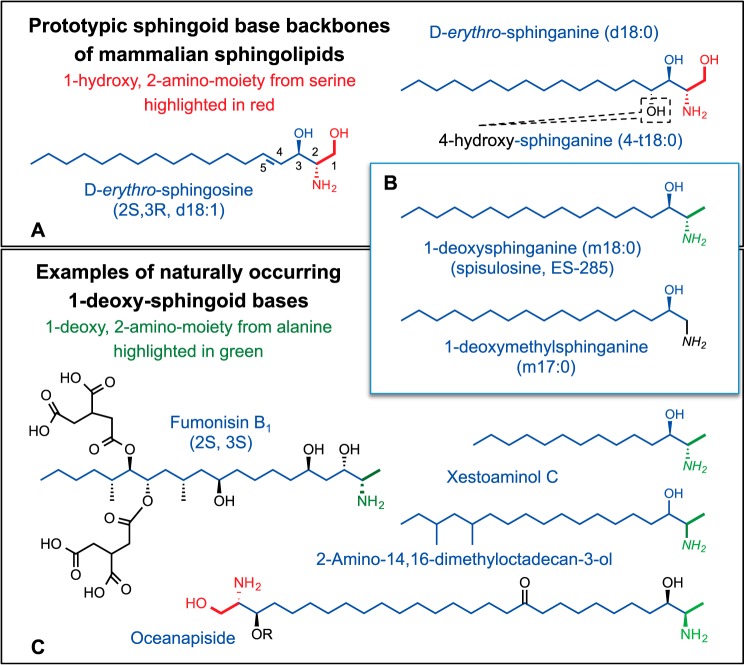
**Representative sphingoid bases and 1-deoxysphingoid bases.** *A*, three of the traditional sphingoid bases of mammalian sphingolipids: sphingosine, sphinganine, and 4-hydroxysphinganine (phytosphingosine). There is also some degree of variation in the alkyl chain length, branching, and number of additional double bonds and hydroxyls (not shown). *B*, two simple 1-deoxy-sphingoid bases that are produced by mammals and other organisms; these are also known to vary in chain length and double bonds. *C*, some of the broader structural variation in 1-deoxy-sphingoid bases produced by other organisms. For all of the panels, these structures have been highlighted in *red* to display the portions that are derived biosynthetically from serine, and in *green* for alanine. Some additional information for the compounds has been given in *parentheses* (stereochemistry and alternative names and abbreviations). For more information, see the text and Ref. 5.

The 2-amino, 1,3-diol moieties of sphingosine (Fig. 1*A*) were first described in a letter to the editors of *The Journal of Biological Chemistry* from H. E. Carter and colleagues in 1942 and then in a full manuscript (1). The term “sphingolipid” was also proposed (2) for this category of compounds, building on the “sphingo-” morpheme chosen by J. L. W. Thudichum in naming “sphingosin” for “ … the many enigmas which it presented to the inquirer … ” (3).

Sphingosine is the prevalent member of a family of traditional sphingoid bases (Fig. 1*A*) found in complex sphingolipids such as ceramide (Cer),^2^ sphingomyelin (SM), glycosphingolipids, etc. that are important for cell structure and signaling (4). Many organisms, such as fungi, bivalves, and sponges, *inter alia*, additionally have 1-deoxysphingolipids (1-deoxySL) (Fig. 1, *B* and *C*) (5), and this minireview provides an introduction to these fascinating compounds, and especially ones now known to be made by mammals (6–8).

## Examples of 1-Deoxy-sphingoid Bases

### Sphinganine Analog Mycotoxins

The most extensively studied 1-deoxy-sphingoid bases are represented by fumonisin B_1_ (FB_1_, Fig. 1*C*). These sphinganine analog mycotoxins are produced by *Fusarium verticillioides* and related fungi (9) that infest maize and cause diseases in plants (10) and in animals that consume contaminated food (11–14). Their major biochemical targets in both plants (10) and animals (11–13, 15) are ceramide synthases (CerS), enzymes responsible for *N*-acylation of sphingoid bases (16, 17). In addition to being inhibitors of CerS, fumonisins are *N*-acylated by CerS (18), as is the aminopentol backbone released from fumonisins when corn is treated with lye in preparation of masa (19). *N*-Acyl-aminopentols also inhibit CerS.

Disruption of sphingolipid metabolism by fumonisins and related AAL toxins (from *Alternaria alternata*) (10) induces plant programmed cell death pathways associated with defense and disease (20, 21). This is thought to be a major reason that these mycotoxins are produced, but they might additionally provide protection against other inhabitants of the ecological niche of these fungi (22).

Fumonisin consumption causes a wide spectrum of animal disease: hepatotoxicity and hepatocarcinogenicity, renal toxicity, neurotoxicity, pulmonary edema (9, 23), and in humans, esophageal cancer (9, 11–14) and probably birth defects (13, 24, 25). It is not surprising that they produce so many disorders because CerS inhibition causes buildup of highly bioactive compounds (sphinganine, sphinganine 1-phosphate, *N*-acetyl-sphinganine, and others) and suppresses biosynthesis of Cer and complex sphingolipids, depending on the length of exposure and dosage (11, 12). FB_1_ is often used as a tool to block Cer production and study Cer functions; however, the results must be interpreted with caution because this alters many other bioactive sphingolipids.

### Oceanin, Calyxin, and Other Complex 1-Deoxy-sphingoid Bases

Perhaps the most structurally amazing 1-deoxy analogs are “two-headed”, *i.e.* appearing as if two sphingoid bases are connected tail-to-tail (see oceanapiside from *Oceanapia phillipensis* (26, 27), Fig. 1*C*). These compounds often display antibacterial or antifungal activity, which might be their biologic function; many are cytotoxic for cancer cells (27–32). These compounds illustrate only a fraction of the sphingoid base biodiversity (5).

### Simple 1-Deoxysphingoid Bases, e.g. 1-Deoxysphinganines and Related Compounds

Many organisms have been known to produce simple 1-deoxy- and 1-deoxymethyl-sphingoid bases (5) (Fig. 1, *B* and *C*) such as xestoaminol C from *Xestospongia* sp.) and a methyl-branched 1-deoxysphinganine(2-amino-14,16-dimethyl-octadecan-3-ol, 2-AOD-3-ol), produced by *Fusarium avenaceum*, a fungus found on grains and fruit (33, 34). 1-Deoxysphinganine (Fig. 1*B*) was initially named spisulosine when isolated from the edible Stimpson's surf clam, or Atlantic surf clam (*Spisula polynyma*), during a screen for anticancer compounds (35). Being cytotoxic for cancer cells in culture (36, 37), it has been evaluated in phase I clinical trials, which will be described later in this minireview.

## Mammalian Production of 1-Deoxysphingoid Bases

Considering the unusual structural features and cytotoxicity of 1-deoxysphingoid bases, it came as a surprise when mammals, including humans, were found to produce them, as shown by two independent lines of investigation published at approximately the same time (6–8). One study discovered that mutations in the initial enzyme of traditional sphingoid base biosynthesis (serine palmitoyltransferase, SPT) that cause hereditary sensory and autonomic neuropathy type I disease (HSAN1) allow SPT to utilize l-Ala and glycine to make 1-deoxysphinganine and 1-(deoxymethyl)sphinganine, respectively (7), which are neurotoxic when added to dorsal root ganglia neuron cultures (8).

The other study (6) characterized 1-deoxysphinganine as a previously noticed (38), but unidentified, compound that accumulates when cells in culture or animals are exposed to FB_1_. It was shown to be produced in substantial amounts from l-Ala by wild-type SPT and was probably overlooked previously because it is mainly present as *N*-acyl-metabolites (*e.g.* 1-deoxydihydroceramides, 1-deoxyDHCer) unless CerS is inhibited.

### Background Information about SPT

**FIGURE 2.**
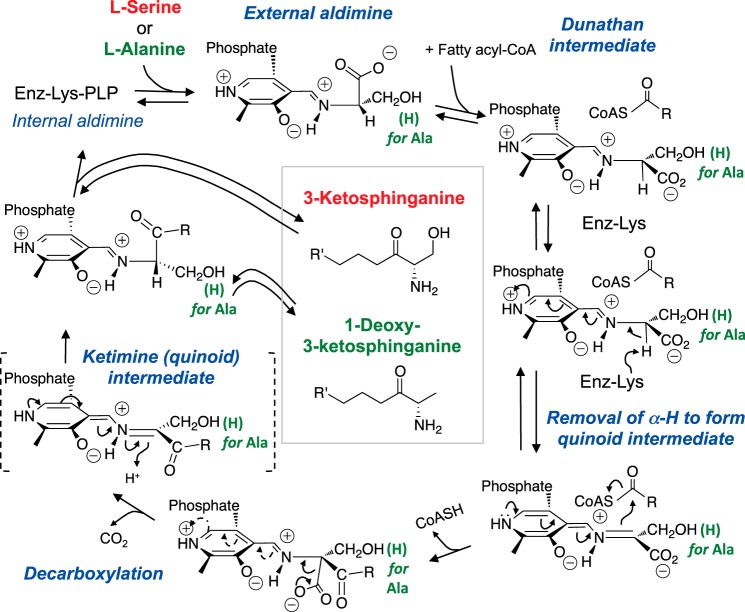
**Scheme for the utilization of l-serine or l-alanine for 3-ketosphingoid base biosynthesis by serine palmitoyltransferase.** This diagram has been modified from Ref. 39 to illustrate the proposed catalytic mechanism for this enzyme and how the intermediates involved in the condensation of l-Ser to make 3-ketosphinganine could plausibly be substituted by l-Ala to make 1-deoxy-3-ketosphinganine with minor variations in the active site chemistry. For all of the panels, these structures have been highlighted in *red* to display the portions that are derived biosynthetically from serine, and in *green* for alanine.

SPT is a family of pyridoxal 5′-phosphate (PLP)-dependent isozymes that catalyze the reaction displayed in Fig. 2 (39, 40). Its proposed mechanism is typical for the α-oxoamine synthase (AOS) family (41), and some of the main features are: formation of a Schiff base between PLP and an active site Lys (called an “internal aldimine”); displacement of Lys when an amino acid substrate is bound (forming the “external aldimine”); orientation of the amino acid-PLP imine in a configuration described as the “Dunathan intermediate” to facilitate abstraction of the amino acid α-proton forming a quinoid intermediate; carbon-carbon bond formation between the amino acid α-carbon and a fatty acyl-CoA, displacing CoASH; decarboxylation of this β-unsaturated intermediate to form a product, external ketimine; protonation of the ketimine to form the external aldimine of the 3-keto-sphingoid base, which is released to regenerate the enzyme PLP internal aldimine. The traditional reaction catalyzed by SPT utilizes l-Ser as the substrate to make 3-ketosphinganine (in *red*), the analogous reaction with l-Ala (*green*) produces 1-deoxysphinganine, and the reaction with glycine produces 1-(deoxymethyl)sphinganine (not shown).

Mammalian SPT appears to be composed of heterotrimeric isozymes that share an SPTLC1 (sometimes referred to as SPT1 or hLCB1) subunit combined with either SPTLC2 (also called SPT2 or hLCB2a) or SPTLC3 (also called SPT3, LBC3, or hLCB2b) subunit and one of two highly related isoforms of a third “small subunit” (in humans, ssSPTa and ssSPTb) (40). The active site Lys (Fig. 2) resides in the SPTLC2/SPTLC3 subunit.

The SPTLC2/SPTLC3 subunit influences the specificity for the acyl-CoA substrate (42) in a manner that also depends on the ssSPT isoform (43, 44). That is, as shown in studies in which cells were transfected with these isoforms in different combinations (43, 44): SPTLC1/SPTLC2/ssSPTa had a clear preference for palmitoyl-CoA (the precursor for the 18-carbon-chain length sphingoid bases); SPTLC1/SPTLC2/ssSPTb utilized both palmitoyl-CoA and stearoyl-CoA (the latter producing 20-carbon-chain length sphingoid bases); SPTLC1/SPTLC3/ssSPTa utilized myristoyl-CoA and palmitoyl-CoA (the former producing 16-carbon-chain length sphingoid bases); and SPTLC1/SPTLC3/ssSPTb seems to use a wide range of chain length fatty acyl-CoAs. Another level of regulation involves ORMDL family proteins, which have been proposed to help control flux through the pathway (45–48).

### Production of 1-Deoxysphingoid Bases by Mutant SPT

HSAN1 neuropathies have been linked to mutations in five different genes, two of which code for SPTLC1 and SPTLC2. These involve missense mutations (49): for SPTLC1, C133W, C133Y, C133R, V144D, A352V, S331F, and S331Y; and for SPTLC2, V359M, G382V, T409M, I504F, A182P, and a more recently reported S384F (50). After the report of elevated 1-deoxySL from l-Ala in studies of the SPTLC1 C133W mutation in humans and transgenic mice (7), 1-deoxySL have been found in other SPT HSAN1 mutations (8, 51).

The kinetic properties of wild-type and mutant SPT have been compared using microsomes from yeast transfected with cDNA for SPTLC1/SPTLC2/ssSPTa *versus* SPTLC1-C133W/SPTLC2/ssSPTa (44), and the major conclusions have been substantiated by studies with CHO-LyB cells, a mammalian cell line with an unstable and inactive SPTLC1 subunit (52). The *K_m_* for l-Ser for mutant SPT was higher than for the wild-type enzyme (∼1.4 *versus* 0.75 mm, respectively), and the *V*_max_ was lower (∼0.3 *versus* 1.4 nmol/mg/min); conversely, the mutant SPT utilized l-Ala better than the wild-type. The *K_m_* and *V*_max_ with l-Ala were ∼9.6 mm and ∼0.1 nmol/mg/min for mutant SPT, and it was difficult to measure the kinetics with l-Ala using the wild-type enzyme. The *K_i_* for l-Ala inhibition of l-Ser utilization was 5 mm for the mutant SPT and 2 mm for wild type. These results suggest that the major effect of this HSAN1 mutation is not to facilitate l-Ala binding but to allow bound l-Ala to react with the acyl-CoA substrate. Although crystal structures are not yet available for mammalian SPT, they have been determined for a soluble homodimeric SPT from *Sphingomonas paucimobilis* EY2395 (53) and were used to map Cys-133 of SPTLC1 onto Asn-100 of the bacterial SPT, which is proximal to the PLP binding site and lies at the dimer interface (39).

A similar approach has been used to analyze V359M, G382V, and I504F mutations in SPTLC2 (54), and all decrease enzyme activity somewhat for reasons that can be rationalized by comparisons with alterations in the soluble enzyme. The impact of these mutations on l-Ala utilization was not reported, but 1-deoxySL have been associated with SPTLC2 mutations A182P (55) and S384F (50). The S384F mutation was suggested to implicate phosphorylation of SPTLC2 at this site as a regulator of 1-deoxySL synthesis by wild-type SPT.

To determine whether lowering 1-deoxySL might be clinically beneficial, Garofalo *et al.* (56) fed a 10% l-Ser-enriched diet to mice bearing a transgene expressing C133W SPTLC1, and 1-deoxySL decreased significantly, reaching the levels of mice with wild-type SPT within 2–4 days. Mice on the l-Ser-enriched diet were also protected from neurodegeneration (measured by mechanical sensitivity and motor performance) and retained neurological function up to 15 months of age; untreated mice developed neuropathy by that age. In contrast to these favorable responses, mice fed a 10% l-Ala diet had elevated 1-deoxySL and developed severe peripheral neuropathy. A pilot study with HSAN1 patients also found that l-Ser supplementation reduced 1-deoxySL levels, and a clinical trial is ongoing (https://clinicaltrials.gov).

### Production of 1-Deoxysphingoid Bases by Wild-type SPT

In the other early study, 1-deoxySL were identified as products of wild-type SPT by mass spectrometry (6), which characterized both the free 1-deoxysphinganine in cells incubated with FB_1_ and the *N*-acyl-derivatives when CerS was not inhibited. This acylation might explain why these compounds have been overlooked previously because they are somewhat difficult to detect in a background of Cer and other neutral lipids.

Wild-type SPT was proven to be the source because biosynthesis of 1-deoxySL from l-Ala was absent in CHO-LyB cells and reappeared when the normal SPT1 subunit was restored (6). The amounts of 1-deoxysphinganine made by wild-type SPT can be quite substantial. For example, LLC-PK1 cells have about half of the level of sphinganine after ∼4 days in culture with FB_1_; Vero cells have high basal 1-deoxySL, which might be due to these cells depleting l-Ser in the medium (57); and RAW264.7 cells (58) have essentially equal amounts of 1-deoxyDHCer and Cer after 4 days in culture, and are also known (59) to deplete the culture medium of l-Ser while accumulating l-Ala and glycine.

There has not yet been an explanation for why wild-type SPT is somewhat “promiscuous” (to use a term applied to mutant SPT) (44) in utilizing three amino acids as substrates, nor whether this might occur with other α-oxoamine synthase family members (40). The side chains of l-Ala and Gly are smaller than the hydroxymethyl group of l-Ser and could fit in the same binding pocket. The favoring of l-Ser appears to be due to an interaction between the side-chain hydroxyl of l-Ser and the 5′-phosphate of PLP, both for substrate binding and for optimal catalytic efficiency (60).

Because amino acid availability is an important factor in the amounts of 1-deoxySL that are made, it would be interesting to know more about other factors that are thought to influence l-Ser utilization for lipid synthesis, such as SERINC (61) and, at least for yeast, CHA1, which codes for an l-Ser deamidase/dehydratase that regulates sphingolipid levels by limiting available l-Ser (and perhaps vice versa) (62).

## Metabolism and Trafficking

### 1-Deoxysphingoid Base Metabolism

**FIGURE 3.**
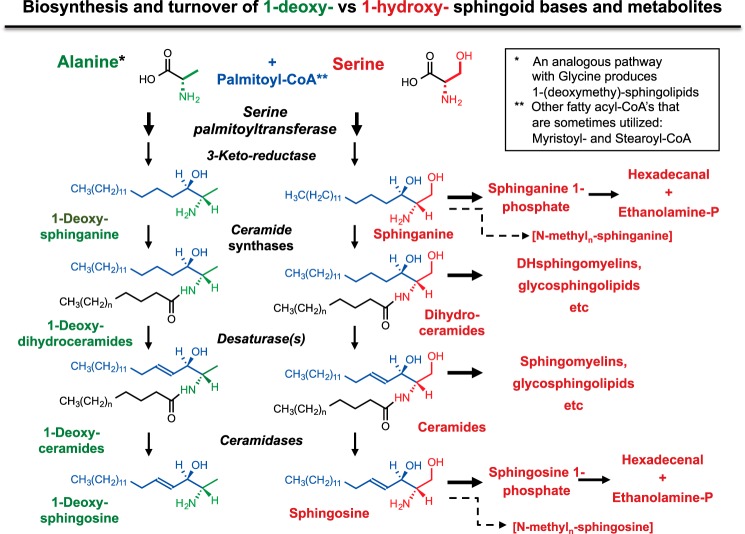
**Abbreviated pathway for the biosynthesis and turnover of 1-deoxy-sphingoid bases and traditional sphingoid bases.** This scheme summarizes the steps of *de novo* biosynthesis of traditional sphingoid bases (sphinganine and sphingosine) in *red*, as well as their turnover via phosphorylation and cleavage. The carbons from palmitate are shown in *blue*. The analogous metabolic steps are shown in *green*, as far as they are thought to occur-for 1-deoxysphinganine (produced from alanine) and 1-(deoxymethyl)sphinganine (produced from glycine, not shown). The *dashed line* indicates the known *N*-methylation of sphingoid bases, which might also occur for 1-deoxy-sphingoid bases, but this has not yet been established. For more information, see the text and Ref. 4.

Most publications on 1-deoxySL have described them as total 1-deoxysphinganines or 1-deoxySL rather than as individual molecular species because they have been quantified after acid hydrolysis to release the free sphingoid bases. As noted above, when specific molecular species are analyzed by LC-MS/MS, the majority of traditional and 1-deoxysphingoid bases are *N*-acyl-derivatives (Fig. 3) (4).

In this pathway, the initially formed 3-keto-intermediates are rapidly reduced and *N*-acylated followed by the addition of a headgroup (dihydroSM, etc.), desaturation to produce the backbone double bond (making Cer), and then the addition of headgroups *or* hydrolyzed to sphingosine, which can be reacylated or converted to sphingosine 1-phosphate (S1P), which is cleaved to ethanolamine phosphate and hexadecenal (hexadecanal from sphinganine 1-phosphate). There are also reports of *N*-methylation of some sphingoid bases (63, 64).

The early steps of this pathway appear to be similar for the 1-deoxysphingoid bases (Fig. 3). The kinetics parameters for *N*-acylation of various sphingoid base variants have been compared using rat liver microsomes (19). The apparent *K_m_* for 1-deoxysphinganine (2 μm) is somewhat higher than for sphinganine (0.5 μm), but the *V*_max_ values are similar. Individual CerS have not been analyzed, but the *N*-acyl-chain length distributions of 1-deoxy(DH)Cer of different types of cells suggest that most or all of the CerS accommodate these compounds (65). Little is known about desaturation of 1-deoxy(DH)Cer; likewise, the possibility of alternative metabolites, such as *N*-methylated species, has not been explored. Turnover by lyase cleavage (Fig. 3) would appear to be unavailable to 1-deoxySL unless S1P lyase, or another enzyme, can catalyze an analogous reaction with 1-deoxysphingoid bases.

### 1-Deoxydihydroceramide Trafficking

The intracellular trafficking of Cer has been studied using analogs with an amide-linked fluorescent fatty acid, such as *N*-(7-nitrobenz-2-oxa-1,3-diazol-4-yl)aminohexanoyl- (C_6_-NBD-) (66). C_6_-NBD-Cer is rapidly taken up by cells in culture, and fluorescence is seen first in multiple intracellular compartments (the plasma membrane, ER, nuclear envelope, and mitochondria), and then the Golgi apparatus becomes intensely fluorescent concomitant with its metabolism to C_6_-NBD-SM and C_6_-NBD-GlcCer, which appear at the plasma membrane after longer times. In contrast, C_6_-NBD-1-deoxyDHCer (67) was neither metabolized nor labeled the Golgi apparatus and plasma membrane, even after prolonged incubation. Thus, the 1-deoxySL do not appear to undergo the typical trafficking of traditional Cer, which is in agreement with similar studies with a 1-methoxy analog (68). The 1-deoxySL in plasma (69, 70) appear to be associated mainly with lipoproteins (71), which might be of hepatic origin (70).

## Cellular Effects of 1-Deoxy-sphingolipids

Some of the earliest findings with 1-deoxysphinganine were that it has diverse effects on cell growth and survival: sometimes stimulating cell proliferation (for Swiss 3T3 cells at 1 μm) (72); sometimes inhibiting growth (for Vero cells at ∼2 μm), possibly due to disruption of actin stress fibers through inactivation of Rho (35), and for the human glioblastoma cell line SHG-44 (73); and often displaying cytotoxicity at low micromolar concentrations for DU145 and LLCPK1 cells (6), MDA MB 468 cells (65), and PC-3 and LNCaP cells (37), as examples. The cytotoxicity has been proposed to have several causes: stimulation of *de novo* synthesis of Cer and PKCζ activation (37); and an atypical cell death program with activation of caspase 3 and 12 and altered phosphorylation of p53 (36). Endoplasmic reticulum stress might also have a role in 1-deoxySL-mediated apoptosis (44, 74). Effects on insulin-producing cells (75) include compromised glucose-stimulated insulin secretion, intracellular accumulation of filamentous actin, activation of Rac1, increased CerS5 expression, and morphologic changes characteristic of senescent, necrotic, and apoptotic cells.

Other reported effects of 1-deoxySL are sphingosine kinase 1 inhibition (and/or its proteasomal degradation) (76) and perturbation of membrane structure because 1-deoxy(DH)Cer are poorly miscible with other lipids (some 1-deoxySL are not even capable of forming monolayers at the air-water interface) (58). The latter might contribute to the formation of lipid bodies in cells accumulating 1-deoxySL (77). Another intriguing finding is that 1-deoxy-(DH)Cer have been reported to be one of the endogenous ligands for human CD1b antigen-presenting molecules (78). As a cautionary note, it is difficult to determine what the normal functions of 1-deoxSL are because studies with cells in culture begin with cells that probably already contain abnormally high 1-deoxySL because they are present in serum and/or produced by the cells themselves, due to the tendency of many cell lines to deplete l-Ser and accumulate l-Ala in the medium.

## 1-Deoxy-sphingoid Bases and Other Disease

### Diabetes

In common with HSAN1, one of the clinical complications of diabetes mellitus is sensory neuropathy; therefore, connections between 1-deoxySL and diabetes have been explored. A case-control study of plasma from healthy and diabetic individuals found that 1-deoxySL levels were higher in the diabetic group, which also displayed lower plasma Ser (71). 1-DeoxySL have been found to be elevated in plasma from subjects with metabolic syndrome (79) and type 2 diabetes (80) (levels in type 1 diabetes did not differ from controls). 1-DeoxySL were also examined as possible predictive biomarkers for type 2 diabetes (81) in a prospective cohort with 339 individuals who were followed for a period of 8 years, and levels were elevated in patients with metabolic syndrome, impaired fasting glucose, and type 2 diabetes and for patients who developed diabetes during the follow-up period. 1-DeoxySL levels were found to be significantly elevated in plasma from patients with distal sensorimotor polyneuropathy, a frequent, disabling complication of diabetes mellitus, and were detectable in early disease stages but did not correlate with the clinical course (82).

In analogy to the studies conducted with an animal model for HSAN1, l-Ser supplementation has been tested in streptozotocin-induced diabetic rats (81). This intervention not only lowered plasma 1-deoxySL but also improved mechanical sensitivity, in agreement with the hypothesis that 1-deoxySL are involved in the pathology of diabetic neuropathy and l-Ser supplementation might be clinically beneficial. It is worth mentioning that plasma l-Ala is elevated following glucose ingestion (83), and fructose ingestion has an even greater effect on plasma l-Ala concentration (84).

### Non-alcoholic Fatty Liver Disease, Especially Non-alcoholic Steatohepatitis (NASH)

Non-alcoholic fatty liver disease is associated with metabolic syndrome and is becoming one of the most common forms of liver disease worldwide. It is thought to progress from relatively benign stages to steatohepatitis (NASH), which can develop into end-stage liver disease, cirrhosis, and sometimes hepatocellular carcinoma. A recent double-blinded study of plasma, liver biopsies, and urinary lipids from 88 subjects with liver histology categorized as normal, steatotic, NASH, or cirrhotic (70) found that a diverse panel of 20 plasma lipids and aqueous metabolites separated these states by linear discriminant analysis, with the compounds that gave the greatest distinction between NASH and steatosis including the 1-deoxyDHCer. A possible explanation for this association might be l-Ser deficiency that has been reported for NASH (85).

### Defective Ser Biosynthesis

l-Ser is made *de novo* by a pathway initiated by d-3-phosphoglycerate dehydrogenase (PHGDH), and mice carrying a brain-specific deletion of *Phgdh* have been used to study the effects of defects in this pathway on 1-deoxySL (77). The mice displayed reductions in both l-Ser and d-Ser and elevation of 1-deoxySL that were associated with mild microcephaly and atrophy of the forebrain, including the cerebral cortex and hippocampus. No significant changes in traditional Cer and SL were noted. Because humans with genetic defects in this enzyme exhibit Ser deficiency and severe neurological symptoms, these results raise the possibility that 1-deoxySL might be involved in the central neurological symptoms (77).

### TNF-dependent Toxicity via Caspase Signaling in Dopaminergic Neurons

Dopaminergic neurons in the ventral midbrain selectively degenerate in Parkinson disease, and TNF can increase neuronal cell death. TNF treatment of dopaminergic neurons has been found to increase 1-deoxySL, which reduce cell viability and inhibit neurite outgrowth and branching in primary dopaminergic neurons when added exogenously (86). Therefore, induction of *de novo* biosynthesis of 1-deoxySL might be involved in the neurotoxicity of TNF for dopaminergic neurons.

## 1-Deoxy-sphingoids as Therapeutic Agents?

### Clinical Trials with Atypical Sphingoid Bases

The first structural variant that was evaluated in a phase I clinical trial (87) was Safingol (l-*threo*-sphinganine), which has 2*S*,3*S* stereochemistry as found in fumonisins (Fig. 1*C*). This is not a 1-deoxySL but inhibits sphingosine kinase and affects some of the same targets as 1-deoxysphinganine (88). The maximum tolerated dose was 840 mg/m^2^ (∼1–2 g based on adult body surface areas of ∼1.5–2 m^2^) administered intravenously over 120 min, with the dose-limiting toxicity attributed to hepatic enzyme elevation. Plasma S1P was reduced for Safingol doses of 750–930 mg/m^2^. Of the 37 patients that were evaluated for response, six were reported to have some degree of disease stabilization, and one patient with adrenal cortical cancer had regression of liver and lung metastases.

Several phase I clinical trials have been conducted with 1-deoxysphinganine (named “ES-285”), and relevant findings from two will be mentioned here. From dose-escalating studies (89, 90), the maximum tolerated dose was ∼200 mg/m^2^ (*i.e.* ∼0.3–0.4 g for body surface areas of 1.5–2 m^2^, respectively). The dose-limiting toxicities were relatively consistent for all the studies: hepatic and neurological toxicity as well as injection site reactions. One patient who received eight infusions of ES-285 at 128 mg/m^2^ developed numbness of the face, hands, and feet that worsened rapidly to neuropathy, pain, and general weakness that was assessed to contribute to his death (90). Clinical development of ES-285 as a single agent was discontinued due to its questionable safety profile and limited antitumor activity. Noteworthy from these trials was the similarity in the adverse effects and the neuropathies that have been associated with elevations in 1-deoxySL produced *de novo*.

### Animal Studies with a Synthetic 1-Deoxy-sphingoid Base

**FIGURE 4.**
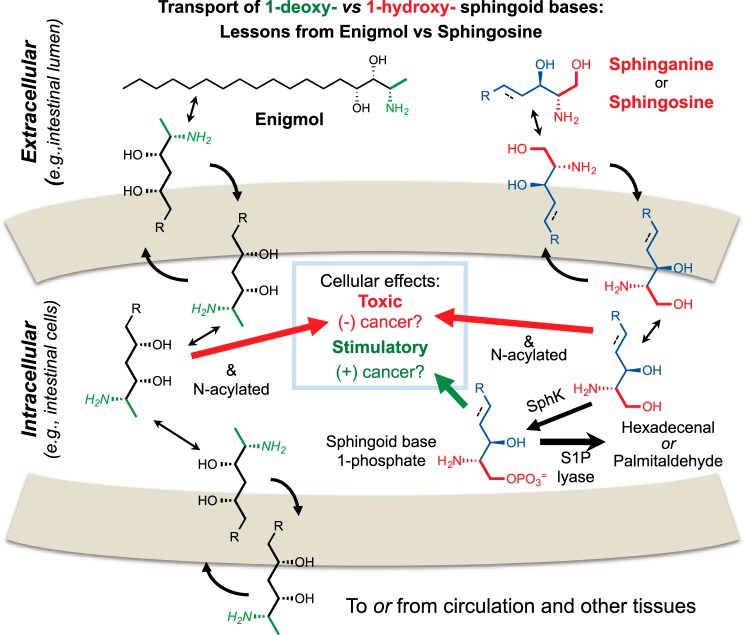
**A schematic representation of the intestinal uptake, metabolism, effects on intestinal cells, and transport to blood and tissues of traditional sphingoid bases (sphingosine and sphinganine) and a synthetic 1-deoxy-sphingoid base (Enigmol).** As shown on the *right*, traditional sphingoid bases are absorbed well from the lumen of the intestinal tract, but most are phosphorylated by sphingosine kinase (SphK) and degraded by S1P lyase. Nutritional studies have shown suppression of colon cancer by dietary sphingolipids, probably through the sphingoid base before phosphorylation and cleavage (94); however, if S1P accumulates (for example, due to defective S1P lyase), this can promote cancer (95). Shown on the *left* are findings with the synthetic 1-deoxy-sphingoid base Enigmol, which is absorbed more efficiently and transferred to blood and tissues, presumably because it cannot undergo phosphorylation and cleavage. Enigmol has also been shown to suppress intestinal tumorigenesis prostate cancer in animal models (91, 92). The color scheme is analogous to the one used in Figs. 1 and 3.

A synthetic 1-deoxysphingoid base, named Enigmol (Fig. 4), displayed tumor suppression with little toxicity when administered to mouse models for colon and prostate cancer (91, 92). Enigmol is not phosphorylated and is poorly *N*-acylated (93), and one of the most interesting findings from these *in vivo* studies was a high oral bioavailability *versus* traditional sphingoid bases. The likely explanation for this difference, which might apply to other 1-deoxy-sphingoid bases, is shown in Fig. 4. Traditional sphingoid bases are readily taken up by intestinal cells but mainly phosphorylated and degraded (94), which limits their effectiveness against colon cancer targets, but cleavage reduces the likelihood that the intermediate S1P will promote carcinogenesis (94, 95). Lacking the 1-hydroxyl group, 1-deoxysphingoid bases (at least as exemplified by Enigmol) are absorbed, escape phosphorylation and degradation, and appear in blood and tissues (91). Another factor affecting the absorption of these compounds is efflux via P-glycoprotein (96). All in all, the possible uptake of 1-deoxySL from food highlights the need for a better understanding of their effect(s) on health.

### Other Clinical Applications?

Sphingolipids and sphingoid base-like compounds, including derivatives of such compounds, have been suggested to offer promise as antibacterial (97) and antifungal (98) drugs and for other diseases (99, 100).

## Conclusions and Future Perspectives

The capacity to make 1-deoxy-sphingoid bases was once thought to be the purview of organisms that make bizarre secondary metabolites, but is now clearly established to be a process shared by mammals, including humans. This leads one to wonder whether these compounds are made as accidents of a sloppy *de novo* biosynthesis pathway or to perform biological functions. Also, considering their widespread occurrence, how much is present in food, and to what extent does the diet affect tissue 1-deoxySL? Because these compounds can be toxic (but possibly beneficial), how many ways do they impact health? These are some of the intriguing mysteries to be solved for this branch of a family of compounds long known for their enigmas.

## Footnotes

2 The abbreviations used are:

Cer: ceramide
1-deoxyDHCer: 1-deoxydihydroceramides (*N*-acyl-1-deoxysphinganine)
1-deoxy(DH)Cer: 1-deoxyceramides and 1-deoxydihydroceramides
1-deoxySL: 1-deoxysphingo-lipids (all compounds lacking 1-hydroxyl on sphingoid base)
CerS: ceramide synthase
DHCer: dihydroceramide
(DH)Cer: ceramides and dihydroceramides
FB_1_: fumonisin B_1_
HSAN1: hereditary sensory and autonomic neuropathy type I disease
PLP: pyridoxal 5′-phosphate
S1P: sphingosine 1-phosphate
SM: sphingomyelin
SPT: serine palmitoyltransferase
NASH: non-alcoholic steatohepatitis
C_6_-NBD: *N*-(7-nitrobenz-2-oxa-1,3-diazol-4-yl)aminohexanoyl
ssSPT: small subunit SPT
h: human.

## References

[1] Carter H. E. Glick F. J. Norris W. P. Phillips G. E. (1947) Biochemistry of the sphingolipides. III. Structure of sphingosine. J. Biol. Chem. 170, 285–294

[2] Carter H. E. Haines W. J. Ledyard W. E. Norris W. P. (1947) Biochemistry of the sphingolipides. I. Preparation of sphingolipides from beef brain and spinal cord. J. Biol. Chem. 169, 77–82 20240540

[3] Thudichum J. L. W. (1881) Researches on the chemical constitution of the non-phosphorized group of nitrogenous principles of the brain. Ann. Chem. Med. 2, 1–53

[4] Merrill A. H. Jr. (2011) Sphingolipid and glycosphingolipid metabolic pathways in the era of sphingolipidomics. Chem. Rev. 111, 6387–6422 21942574 10.1021/cr2002917 PMC3191729

[5] Pruett S. T. Bushnev A. Hagedorn K. Adiga M. Haynes C. A. Sullards M. C. Liotta D. C. Merrill A. H. Jr. (2008) Biodiversity of sphingoid bases (“sphingosines”) and related amino alcohols. J. Lipid Res. 49, 1621–1639 18499644 10.1194/jlr.R800012-JLR200 PMC2444003

[6] Zitomer N. C. Mitchell T. Voss K. A. Bondy G. S. Pruett S. T. Garnier-Amblard E. C. Liebeskind L. S. Park H. Wang E. Sullards M. C. Merrill A. H. Jr. Riley R. T. (2009) Ceramide synthase inhibition by fumonisin B1 causes accumulation of 1-deoxysphinganine: a novel category of bioactive 1-deoxysphingoid bases and 1-deoxydihydroceramides biosynthesized by mammalian cell lines and animals. J. Biol. Chem. 284, 4786–4795 19095642 10.1074/jbc.M808798200 PMC2643501

[7] Eichler F. S. Hornemann T. McCampbell A. Kuljis D. Penno A. Vardeh D. Tamrazian E. Garofalo K. Lee H. J. Kini L. Selig M. Frosch M. Gable K. von Eckardstein A. Woolf C. J. Guan G. Harmon J. M. Dunn T. M. Brown R. H. Jr. (2009) Overexpression of the wild-type SPT1 subunit lowers desoxysphingolipid levels and rescues the phenotype of HSAN1. J. Neurosci. 29, 14646–14651 19923297 10.1523/JNEUROSCI.2536-09.2009 PMC3849752

[8] Penno A. Reilly M. M. Houlden H. Laurá M. Rentsch K. Niederkofler V. Stoeckli E. T. Nicholson G. Eichler F. Brown R. H. Jr. von Eckardstein A. Hornemann T. (2010) Hereditary sensory neuropathy type 1 is caused by the accumulation of two neurotoxic sphingolipids. J. Biol. Chem. 285, 11178–11187 20097765 10.1074/jbc.M109.092973 PMC2856995

[9] Marasas W. F. (2001) Discovery and occurrence of the fumonisins: a historical perspective. Environ. Health Perspect. 109, Suppl. 2, 239–243 11359691 10.1289/ehp.01109s2239 PMC1240671

[10] Abbas H. K. Duke S. O. Merrill A. H. Jr. Wang E. Shier W. T. (1998) Phytotoxicity of australifungin, AAL-toxins and fumonisin B_1_ to *Lemna pausicostata*. Phytochemistry 47, 1509–1514

[11] Merrill A. H. Jr. Sullards M. C. Wang E. Voss K. A. Riley R. T. (2001) Sphingolipid metabolism: roles in signal transduction and disruption by fumonisins. Environ. Health Perspect. 109, Suppl. 2, 283–289 11359697 10.1289/ehp.01109s2283 PMC1240677

[12] Riley R. T. Enongene E. Voss K. A. Norred W. P. Meredith F. I. Sharma R. P. Spitsbergen J. Williams D. E. Carlson D. B. Merrill A. H. Jr. (2001) Sphingolipid perturbations as mechanisms for fumonisin carcinogenesis. Environ. Health Perspect. 109, Suppl. 2, 301–308 11359699 10.1289/ehp.01109s2301 PMC1240679

[13] Suarez L. Felkner M. Brender J. D. Canfield M. Zhu H. Hendricks K. A. (2012) Neural tube defects on the Texas-Mexico border: what we've learned in the 20 years since the Brownsville cluster. Birth Defects Res. A Clin. Mol. Teratol. 94, 882–892 22945287 10.1002/bdra.23070

[14] Wu F. Groopman J. D. Pestka J. J. (2014) Public health impacts of foodborne mycotoxins. Annu. Rev. Food Sci. Technol. 5, 351–372 24422587 10.1146/annurev-food-030713-092431

[15] Wang E. Norred W. P. Bacon C. W. Riley R. T. Merrill A. H. Jr. (1991) Inhibition of sphingolipid biosynthesis by fumonisins: implications for diseases associated with *Fusarium moniliforme*. J. Biol. Chem. 266, 14486–14490 1860857

[16] Tidhar R. Futerman A. H. (2013) The complexity of sphingolipid biosynthesis in the endoplasmic reticulum. Biochim. Biophys. Acta 1833, 2511–2518 23611790 10.1016/j.bbamcr.2013.04.010

[17] Venkataraman K. Riebeling C. Bodennec J. Riezman H. Allegood J. C. Sullards M. C. Merrill A. H. Jr. Futerman A. H. (2002) Upstream of growth and differentiation factor 1 (*uog1*), a mammalian homolog of the yeast longevity assurance gene 1 (*LAG1*), regulates *N*-stearoyl-sphinganine (C18-(dihydro)ceramide) synthesis in a fumonisin B1-independent manner in mammalian cells. J. Biol. Chem. 277, 35642–35649 12105227 10.1074/jbc.M205211200

[18] Harrer H. Humpf H. U. Voss K. A. (2015) *In vivo* formation of *N*-acyl-fumonisin B1. Mycotoxin Res. 31, 33–40 25326150 10.1007/s12550-014-0211-5 PMC4298654

[19] Humpf H. U. Schmelz E. M. Meredith F. I. Vesper H. Vales T. R. Wang E. Menaldino D. S. Liotta D. C. Merrill A. H. Jr. (1998) Acylation of naturally occurring and synthetic 1-deoxysphinganines by ceramide synthase: formation of *N*-palmitoyl-aminopentol produces a toxic metabolite of hydrolyzed fumonisin, AP1, and a new category of ceramide synthase inhibitor. J. Biol. Chem. 273, 19060–19064 9668088 10.1074/jbc.273.30.19060

[20] Gilchrist D. G. (1997) Mycotoxins reveal connections between plants and animals in apoptosis and ceramide signaling. Cell Death Differ. 4, 689–698 16465281 10.1038/sj.cdd.4400312

[21] Berkey R. Bendigeri D. Xiao S. (2012) Sphingolipids and plant defense/disease: the “death” connection and beyond. Front. Plant Sci. 3, 68 22639658 10.3389/fpls.2012.00068 PMC3355615

[22] Fox E. M. Howlett B. J. (2008) Secondary metabolism: regulation and role in fungal biology. Curr. Opin. Microbiol. 11, 481–487 18973828 10.1016/j.mib.2008.10.007

[23] Voss K. A. Riley R. T. Norred W. P. Bacon C. W. Meredith F. I. Howard P. C. Plattner R. D. Collins T. F. Hansen D. K. Porter J. K. (2001) An overview of rodent toxicities: liver and kidney effects of fumonisins and *Fusarium moniliforme*. Environ. Health Perspect. 109, Suppl. 2, 259–266 11359694 10.1289/ehp.01109s2259 PMC1240674

[24] Marasas W. F. Riley R. T. Hendricks K. A. Stevens V. L. Sadler T. W. Gelineau-van Waes J. Missmer S. A. Cabrera J. Torres O. Gelderblom W. C. Allegood J. Martínez C. Maddox J. Miller J. D. Starr L. Sullards M. C. Roman A. V. Voss K. A. Wang E. Merrill A. H. Jr. (2004) Fumonisins disrupt sphingolipid metabolism, folate transport, and neural tube development in embryo culture and *in vivo*: a potential risk factor for human neural tube defects among populations consuming fumonisin-contaminated maize. J. Nutr. 134, 711–716 15051815 10.1093/jn/134.4.711

[25] Gelineau-van Waes J. Voss K. A. Stevens V. L. Speer M. C. Riley R. T. (2009) Maternal fumonisin exposure as a risk factor for neural tube defects. Adv. Food Nutr. Res. 56, 145–181 19389609 10.1016/S1043-4526(08)00605-0

[26] Nicholas G. M. Molinski T. F. (2000) Enantiodivergent biosynthesis of the dimeric sphingolipid oceanapiside from the marine sponge *Oceanapia phillipensis*: determination of remote stereochemistry. J. Am. Chem. Soc. 122, 4011–4019

[27] Makarieva T. N. Dmitrenok P. S. Zakharenko A. M. Denisenko V. A. Guzii A. G. Li R. Skepper C. K. Molinski T. F. Stonik V. A. (2007) Rhizochalins C and D from the sponge *Rhizochalina incrustata*: a rare *threo*-sphingolipid and a facile method for determination of the carbonyl position in α,ω-bifunctionalized ketosphingolipids. J. Nat. Prod. 70, 1991–1998 18052325 10.1021/np0704811

[28] Zhou B.-N. Mattern M. P. Johnson R. K. Kingston D. G. I. (2001) Structure and stereochemistry of a novel bioactive sphingolipid from a *Calyx* sp. Tetrahedron 57, 9549–9554

[29] Nicholas G. M. Li R. MacMillan J. B. Molinski T. F. (2002) Antifungal Activity of bifunctional sphingolipids: intramolecular synergism within long-chain α,ω-bis-aminoalcohols. Bioorg. Med. Chem. Lett. 12, 2159–2162 12127527 10.1016/s0960-894x(02)00367-0

[30] Makarieva T. N. Guzii A. G. Denisenko V. A. Dmitrenok P. S. Santalova E. A. Pokanevich E. V. Molinski T. F. Stonik V. A. (2005) Rhizochalin A, a novel two-headed sphingolipid from the sponge *Rhizochalina incrustata*. J. Nat. Prod. 68, 255–257 15730256 10.1021/np049710z

[31] Crews P. Clark D. P. Tenney K. (2003) Variation in the alkaloids among Indo-Pacific *Leucetta* sponges. J. Nat. Prod. 66, 177–182 12608847 10.1021/np020371o

[32] Willis R. H. de Vries D. J. (1997) BRS1, a C30 bis-amino, bis-hydroxy polyunsaturated lipid from an Australian calcareous sponge that inhibits protein kinase C. Toxicon 35, 1125–1129 9248010 10.1016/s0041-0101(96)00218-8

[33] Uhlig S. Petersen D. Flåøyen A. Wilkins A. (2005) 2-Amino-14,16-dimethyloctadecan-3-ol, a new sphingosine analogue toxin in the fungal genus *Fusarium*. Toxicon 46, 513–522 16084551 10.1016/j.toxicon.2005.06.016

[34] Sørensen J. L. Phipps R. K. Nielsen K. F. Schroers H. J. Frank J. Thrane U. (2009) Analysis of *Fusarium avenaceum* metabolites produced during wet apple core rot. J. Agric. Food Chem. 57, 1632–1639 19170495 10.1021/jf802926u

[35] Cuadros R. Montejo de Garcini E. Wandosell F. Faircloth G. Fernández-Sousa J. M. Avila J. (2000) The marine compound spisulosine, an inhibitor of cell proliferation, promotes the disassembly of actin stress fibers. Cancer Lett. 152, 23–29 10754202 10.1016/s0304-3835(99)00428-0

[36] Salcedo M. Cuevas C. Alonso J. L. Otero G. Faircloth G. Fernandez-Sousa J. M. Avila J. Wandosell F. (2007) The marine sphingolipid-derived compound ES 285 triggers an atypical cell death pathway. Apoptosis 12, 395–409 17191124 10.1007/s10495-006-0573-z

[37] Sánchez A. M. Malagarie-Cazenave S. Olea N. Vara D. Cuevas C. Díaz-Laviada I. (2008) Spisulosine (ES-285) induces prostate tumor PC-3 and LNCaP cell death by *de novo* synthesis of ceramide and PKCζ activation. Eur. J. Pharmacol. 584, 237–245 18343365 10.1016/j.ejphar.2008.02.011

[38] Riley R. T. Voss K. A. Norred W. P. Sharma R. P. Wang E. Merrill A. H. (1998) Fumonisins: mechanism of mycotoxicity. Rev. Med. Vet. 149, 617–626

[39] Raman M. C. Johnson K. A. Yard B. A. Lowther J. Carter L. G. Naismith J. H. Campopiano D. J. (2009) The external aldimine form of serine palmitoyltransferase: structural, kinetic, and spectroscopic analysis of the wild-type enzyme and HSAN1 mutant mimics. J. Biol. Chem. 284, 17328–17339 19376777 10.1074/jbc.M109.008680 PMC2719368

[40] Lowther J. Naismith J. H. Dunn T. M. Campopiano D. J. (2012) Structural, mechanistic and regulatory studies of serine palmitoyltransferase. Biochem. Soc. Trans. 40, 547–554 22616865 10.1042/BST20110769

[41] Eliot A. C. Kirsch J. F. (2004) Pyridoxal phosphate enzymes: mechanistic, structural, and evolutionary considerations. Annu. Rev. Biochem. 73, 383–415 15189147 10.1146/annurev.biochem.73.011303.074021

[42] Hornemann T. Penno A. Rütti M. F. Ernst D. Kivrak-Pfiffner F. Rohrer L. von Eckardstein A. (2009) The SPTLC3 subunit of serine palmitoyltransferase generates short chain sphingoid bases. J. Biol. Chem. 284, 26322–26330 19648650 10.1074/jbc.M109.023192 PMC2785320

[43] Han G. Gupta S. D. Gable K. Niranjanakumari S. Moitra P. Eichler F. Brown R. H. Jr. Harmon J. M. Dunn T. M. (2009) Identification of small subunits of mammalian serine palmitoyltransferase that confer distinct acyl-CoA substrate specificities. Proc. Natl. Acad. Sci. U.S.A. 106, 8186–8191 19416851 10.1073/pnas.0811269106 PMC2688822

[44] Gable K. Gupta S. D. Han G. Niranjanakumari S. Harmon J. M. Dunn T. M. (2010) A disease-causing mutation in the active site of serine palmitoyltransferase causes catalytic promiscuity. J. Biol. Chem. 285, 22846–22852 20504773 10.1074/jbc.M110.122259 PMC2906276

[45] Siow D. Sunkara M. Morris A. Wattenberg B. (2015) Regulation of *de novo* sphingolipid biosynthesis by the ORMDL proteins and sphingosine kinase-1. Adv. Biol. Regul. 57, 42–54 25319495 10.1016/j.jbior.2014.09.002

[46] Gupta S. D. Gable K. Alexaki A. Chandris P. Proia R. L. Dunn T. M. Harmon J. M. (2015) Expression of the ORMDLS, modulators of serine palmitoyltransferase, is regulated by sphingolipids in mammalian cells. J. Biol. Chem. 290, 90–98 25395622 10.1074/jbc.M114.588236 PMC4281770

[47] Kiefer K. Carreras-Sureda A. García-López R. Rubio-Moscardó F. Casas J. Fabriàs G. Vicente R. (2015) Coordinated regulation of the orosomucoid-like gene family expression controls *de novo* ceramide synthesis in mammalian cells. J. Biol. Chem. 290, 2822–2830 25519910 10.1074/jbc.M114.595116 PMC4317021

[48] Oyeniran C. Sturgill J. L. Hait N. C. Huang W. C. Avni D. Maceyka M. Newton J. Allegood J. C. Montpetit A. Conrad D. H. Milstien S. Spiegel S. (2015) Aberrant ORM (yeast)-like protein isoform 3 (ORMDL3) expression dysregulates ceramide homeostasis in cells and ceramide exacerbates allergic asthma in mice. J. Allergy Clin. Immunol. 10.1016/j.jaci.2015.02.031 PMC4591101 25842287

[49] Astudillo L. Sabourdy F. Therville N. Bode H. Ségui B. Andrieu-Abadie N. Hornemann T. Levade T. (2015) Human genetic disorders of sphingolipid biosynthesis. J. Inherit. Metab. Dis. 38, 65–76 25141825 10.1007/s10545-014-9736-1

[50] Ernst D. Murphy S. M. Sathiyanadan K. Wei Y. Othman A. Laurá M. Liu Y. T. Penno A. Blake J. Donaghy M. Houlden H. Reilly M. M. Hornemann T. (2015) Novel HSAN1 mutation in serine palmitoyltransferase resides at a putative phosphorylation site that is involved in regulating substrate specificity. Neuromolecular Med. 17, 47–57 25567748 10.1007/s12017-014-8339-1 PMC4326654

[51] Auer-Grumbach M. Bode H. Pieber T. R. Schabhüttl M. Fischer D. Seidl R. Graf E. Wieland T. Schuh R. Vacariu G. Grill F. Timmerman V. Strom T. M. Hornemann T. (2013) Mutations at Ser331 in the HSN type I gene SPTLC1 are associated with a distinct syndromic phenotype. Eur. J. Med. Genet. 56, 266–269 23454272 10.1016/j.ejmg.2013.02.002 PMC3682180

[52] Momin A. A. Park H. Allegood J. C. Leipelt M. Kelly S. L. Merrill A. H. Jr. Hanada K. (2009) Characterization of mutant serine palmitoyltransferase 1 in LY-B cells. Lipids 44, 725–732 19536577 10.1007/s11745-009-3316-4 PMC4638125

[53] Yard B. A. Carter L. G. Johnson K. A. Overton I. M. Dorward M. Liu H. McMahon S. A. Oke M. Puech D. Barton G. J. Naismith J. H. Campopiano D. J. (2007) The structure of serine palmitoyltransferase: gateway to sphingolipid biosynthesis. J. Mol. Biol. 370, 870–886 17559874 10.1016/j.jmb.2007.04.086

[54] Beattie A. E. Gupta S. D. Frankova L. Kazlauskaite A. Harmon J. M. Dunn T. M. Campopiano D. J. (2013) The pyridoxal 5′-phosphate (PLP)-dependent enzyme serine palmitoyltransferase (SPT): effects of the small subunits and insights from bacterial mimics of human hLCB2a HSAN1 mutations. Biomed. Res. Int. 2013, 194371 24175284 10.1155/2013/194371 PMC3794620

[55] Murphy S. M. Ernst D. Wei Y. Laurà M. Liu Y. T. Polke J. Blake J. Winer J. Houlden H. Hornemann T. Reilly M. M. (2013) Hereditary sensory and autonomic neuropathy type 1 (HSANI) caused by a novel mutation in *SPTLC2*. Neurology 80, 2106–2111 23658386 10.1212/WNL.0b013e318295d789 PMC3716354

[56] Garofalo K. Penno A. Schmidt B. P. Lee H. J. Frosch M. P. von Eckardstein A. Brown R. H. Hornemann T. Eichler F. S. (2011) Oral l-serine supplementation reduces production of neurotoxic deoxysphingolipids in mice and humans with hereditary sensory autonomic neuropathy type 1. J. Clin. Invest. 121, 4735–4745 22045570 10.1172/JCI57549 PMC3225995

[57] Quesney S. Marc A. Gerdil C. Gimenez C. Marvel J. Richard Y. Meignier B. (2003) Kinetics and metabolic specificities of Vero cells in bioreactor cultures with serum-free medium. Cytotechnology 42, 1–11 19002923 10.1023/A:1026185615650 PMC3449507

[58] Jiménez-Rojo N. Sot J. Busto J. V. Shaw W. A. Duan J. Merrill A. H. Jr. Alonso A. Goñi F. M. (2014) Biophysical properties of novel 1-deoxy-(dihydro)ceramides occurring in mammalian cells. Biophys. J. 107, 2850–2859 25517151 10.1016/j.bpj.2014.10.010 PMC4269796

[59] Sakagami H. Kishino K. Amano O. Kanda Y. Kunii S. Yokote Y. Oizumi H. Oizumi T. (2009) Cell death induced by nutritional starvation in mouse macrophage-like RAW264.7 cells. Anticancer Res. 29, 343–347 19331171

[60] Beattie A. E. Clarke D. J. Wadsworth J. M. Lowther J. Sin H. L. Campopiano D. J. (2013) Reconstitution of the pyridoxal 5′-phosphate (PLP) dependent enzyme serine palmitoyltransferase (SPT) with pyridoxal reveals a crucial role for the phosphate during catalysis. Chem. Commun. (Camb.) 49, 7058–7060, 10.1039/c3cc43001d 23814788

[61] Inuzuka M. Hayakawa M. Ingi T. (2005) Serinc, an activity-regulated protein family, incorporates serine into membrane lipid synthesis. J. Biol. Chem. 280, 35776–35783 16120614 10.1074/jbc.M505712200

[62] Montefusco D. J. Newcomb B. Gandy J. L. Brice S. E. Matmati N. Cowart L. A. Hannun Y. A. (2012) Sphingoid bases and the serine catabolic enzyme CHA1 define a novel feedforward/feedback mechanism in the response to serine availability. J. Biol. Chem. 287, 9280–9289 22277656 10.1074/jbc.M111.313445 PMC3308826

[63] Morales P. R. Dillehay D. L. Moody S. J. Pallas D. C. Pruett S. Allgood J. C. Symolon H. Merrill A. H. Jr. (2007) Safingol toxicology after oral administration to TRAMP mice: demonstration of safingol uptake and metabolism by *N*-acylation and *N*-methylation. Drug Chem. Toxicol. 30, 197–216 17613006 10.1080/01480540701375018

[64] Chen Y. J. Hill S. Huang H. Taraboletti A. Cho K. Gallo R. Manchester M. Shriver L. P. Patti G. J. (2014) Inflammation triggers production of dimethylsphingosine from oligodendrocytes. Neuroscience 279, 113–121 25151189 10.1016/j.neuroscience.2014.08.011 PMC12295769

[65] Abad J. L. Nieves I. Rayo P. Casas J. Fabriàs G. Delgado A. (2013) Straightforward access to spisulosine and 4,5-dehydrospisulosine stereoisomers: probes for profiling ceramide synthase activities in intact cells. J. Org. Chem. 78, 5858–5866 23679346 10.1021/jo400440z

[66] Pagano R. E. (1990) The Golgi apparatus: insights from lipid biochemistry. Biochem. Soc. Trans. 18, 361–366 2197129 10.1042/bst0180361

[67] Kok J. W. Nikolova-Karakashian M. Klappe K. Alexander C. Merrill A. H. Jr. (1997) Dihydroceramide biology: structure-specific metabolism and intracellular localization. J. Biol. Chem. 272, 21128–21136 9261117 10.1074/jbc.272.34.21128

[68] Pütz U. Schwarzmann G. (1995) Golgi staining by two fluorescent ceramide analogues in cultured fibroblasts requires metabolism. Eur. J. Cell Biol. 68, 113–121 8575458

[69] Quehenberger O. Armando A. M. Brown A. H. Milne S. B. Myers D. S. Merrill A. H. Bandyopadhyay S. Jones K. N. Kelly S. Shaner R. L. Sullards C. M. Wang E. Murphy R. C. Barkley R. M. Leiker T. J. Raetz C. R. Guan Z. Laird G. M. Six D. A. Russell D. W. McDonald J. G. Subramaniam S. Fahy E. Dennis E. A. (2010) Lipidomics reveals a remarkable diversity of lipids in human plasma. J. Lipid Res. 51, 3299–3305 20671299 10.1194/jlr.M009449 PMC2952570

[70] Gorden D. L. Myers D. S. Ivanova P. T. Fahy E. Maurya M. R. Gupta S. Min J. Spann N. J. McDonald J. G. Kelly S. L. Duan J. Sullards M. C. Leiker T. J. Barkley R. M. Quehenberger O. Armando A. M. Milne S. B. Mathews T. P. Armstrong M. D. Li C. Melvin W. V. Clements R. H. Washington M. K. Mendonsa A. M. Witztum J. L. Guan Z. Glass C. K. Murphy R. C. Dennis E. A. Merrill A. H. Jr. Russell D. W. Subramaniam S. Brown H. A. (2015) Biomarkers of NAFLD progression: a lipidomics approach to an epidemic. J. Lipid Res. 56, 722–736 25598080 10.1194/jlr.P056002 PMC4340319

[71] Bertea M. Rütti M. F. Othman A. Marti-Jaun J. Hersberger M. von Eckardstein A. Hornemann T. (2010) Deoxysphingoid bases as plasma markers in diabetes mellitus. Lipids Health Dis. 9, 84 20712864 10.1186/1476-511X-9-84 PMC2931514

[72] Schroeder J. J. Crane H. M. Xia J. Liotta D. C. Merrill A. H. Jr. (1994) Disruption of sphingolipid metabolism and stimulation of DNA synthesis by fumonisin B1: a molecular mechanism for carcinogenesis associated with *Fusarium moniliforme*. J. Biol. Chem. 269, 3475–3481 8106389

[73] Chen B. S. Yang L. H. Ye J. L. Huang T. Ruan Y. P. Fu J. Huang P. Q. (2011) Diastereoselective synthesis and bioactivity of long-chain anti-2-amino-3-alkanols. Eur. J. Med. Chem. 46, 5480–5486 21955681 10.1016/j.ejmech.2011.09.010

[74] Myers S. J. Malladi C. S. Hyland R. A. Bautista T. Boadle R. Robinson P. J. Nicholson G. A. (2014) Mutations in the *SPTLC1* protein cause mitochondrial structural abnormalities and endoplasmic reticulum stress in lymphoblasts. DNA Cell Biol. 33, 399–407 24673574 10.1089/dna.2013.2182

[75] Zuellig R. A. Hornemann T. Othman A. Hehl A. B. Bode H. Güntert T. Ogunshola O. O. Saponara E. Grabliauskaite K. Jang J. H. Ungethuem U. Wei Y. von Eckardstein A. Graf R. Sonda S. (2014) Deoxysphingolipids, novel biomarkers for type 2 diabetes, are cytotoxic for insulin-producing cells. Diabetes 63, 1326–1339 24379346 10.2337/db13-1042

[76] Byun H. S. Pyne S. Macritchie N. Pyne N. J. Bittman R. (2013) Novel sphingosine-containing analogues selectively inhibit sphingosine kinase (SK) isozymes, induce SK1 proteasomal degradation and reduce DNA synthesis in human pulmonary arterial smooth muscle cells. Medchemcomm 4, 10.1039/C3MD00201B PMC3880124 24396570

[77] Esaki K. Sayano T. Sonoda C. Akagi T. Suzuki T. Ogawa T. Okamoto M. Yoshikawa T. Hirabayashi Y. Furuya S. (2015) l-Serine deficiency elicits intracellular accumulation of cytotoxic deoxy-sphingolipids and lipid body formation. J. Biol. Chem. 290, 14595–14609 25903138 10.1074/jbc.M114.603860 PMC4505526

[78] Huang S. Cheng T. Y. Young D. C. Layre E. Madigan C. A. Shires J. Cerundolo V. Altman J. D. Moody D. B. (2011) Discovery of deoxyceramides and diacylglycerols as CD1b scaffold lipids among diverse groove-blocking lipids of the human CD1 system. Proc. Natl. Acad. Sci. U.S.A. 108, 19335–19340 22087000 10.1073/pnas.1112969108 PMC3228429

[79] Othman A. Rütti M. F. Ernst D. Saely C. H. Rein P. Drexel H. Porretta-Serapiglia C. Lauria G. Bianchi R. von Eckardstein A. Hornemann T. (2012) Plasma deoxysphingolipids: a novel class of biomarkers for the metabolic syndrome? Diabetologia 55, 421–431 22124606 10.1007/s00125-011-2384-1

[80] Wei N. Pan J. Pop-Busui R. Othman A. Alecu I. Hornemann T. Eichler F. S. (2014) Altered sphingoid base profiles in type 1 compared to type 2 diabetes. Lipids Health Dis. 13, 161 25305670 10.1186/1476-511X-13-161 PMC4271467

[81] Othman A. Bianchi R. Alecu I. Wei Y. Porretta-Serapiglia C. Lombardi R. Chiorazzi A. Meregalli C. Oggioni N. Cavaletti G. Lauria G. von Eckardstein A. Hornemann T. (2015) Lowering plasma 1-deoxysphingolipids improves neuropathy in diabetic rats. Diabetes 64, 1035–1045 25277395 10.2337/db14-1325

[82] Dohrn M. F. Othman A. Hirshman S. K. Bode H. Alecu I. Fahndrich E. Karges W. Weis J. Schulz J. B. Hornemann T. Claeys K. G. (2015) Elevation of plasma 1-deoxy-sphingolipids in type 2 diabetes mellitus: a susceptibility to neuropathy? Eur. J. Neurol. 22, 806-e55, 10.1111/ene.12663 25623782

[83] Donatelli M. Russo V. Bucalo M. L. Scarpinato A. Veronelli A. Craveri A. (1992) Plasma alanine and lactate concentrations following glucose ingestion in normal and NIDDM subjects. Diabetes Res. 20, 121–126 1345005

[84] Fukagawa N. K. Veirs H. Langeloh G. (1995) Acute effects of fructose and glucose ingestion with and without caffeine in young and old humans. Metabolism 44, 630–638 7752912 10.1016/0026-0495(95)90121-3

[85] Mardinoglu A. Agren R. Kampf C. Asplund A. Uhlen M. Nielsen J. (2014) Genome-scale metabolic modelling of hepatocytes reveals serine deficiency in patients with non-alcoholic fatty liver disease. Nat. Commun. 5, 3083 24419221 10.1038/ncomms4083

[86] Martinez T. N. Chen X. Bandyopadhyay S. Merrill A. H. Tansey M. G. (2012) Ceramide sphingolipid signaling mediates Tumor Necrosis Factor (TNF)-dependent toxicity via caspase signaling in dopaminergic neurons. Mol. Neurodegener. 7, 45 22973882 10.1186/1750-1326-7-45 PMC3472284

[87] Dickson M. A. Carvajal R. D. Merrill A. H. Jr. Gonen M. Cane L. M. Schwartz G. K. (2011) A phase I clinical trial of safingol in combination with cisplatin in advanced solid tumors. Clin. Cancer Res. 17, 2484–2492 21257722 10.1158/1078-0432.CCR-10-2323 PMC3078945

[88] Coward J. Ambrosini G. Musi E. Truman J. P. Haimovitz-Friedman A. Allegood J. C. Wang E. Merrill A. H. Jr. Schwartz G. K. (2009) Safingol (l-*threo*-sphinganine) induces autophagy in solid tumor cells through inhibition of PKC and the PI3-kinase pathway. Autophagy 5, 184–193 19098447 10.4161/auto.5.2.7361

[89] Massard C. Salazar R. Armand J. P. Majem M. Deutsch E. García M. Oaknin A. Fernández-García E. M. Soto A. Soria J. C. (2012) Phase I dose-escalating study of ES-285 given as a three-hour intravenous infusion every three weeks in patients with advanced malignant solid tumors. Invest. New Drugs 30, 2318–2326 22215532 10.1007/s10637-011-9772-8

[90] Schöffski P. Dumez H. Ruijter R. Miguel-Lillo B. Soto-Matos A. Alfaro V. Giaccone G. (2011) Spisulosine (ES-285) given as a weekly three-hour intravenous infusion: results of a phase I dose-escalating study in patients with advanced solid malignancies. Cancer Chemother. Pharmacol. 68, 1397–1403 21465314 10.1007/s00280-011-1612-1

[91] Symolon H. Bushnev A. Peng Q. Ramaraju H. Mays S. G. Allegood J. C. Pruett S. T. Sullards M. C. Dillehay D. L. Liotta D. C. Merrill A. H. Jr. (2011) Enigmol: a novel sphingolipid analogue with anticancer activity against cancer cell lines and *in vivo* models for intestinal and prostate cancer. Mol. Cancer Ther. 10, 648–657 21398423 10.1158/1535-7163.MCT-10-0754 PMC5536251

[92] Garnier-Amblard E. C. Mays S. G. Arrendale R. F. Baillie M. T. Bushnev A. S. Culver D. G. Evers T. J. Holt J. J. Howard R. B. Liebeskind L. S. Menaldino D. S. Natchus M. G. Petros J. A. Ramaraju H. Reddy G. P. Liotta D. C. (2011) Novel synthesis and biological evaluation of enigmols as therapeutic agents for treating prostate cancer. ACS Med. Chem. Lett. 2, 438–443 24900327 10.1021/ml2000164 PMC4017995

[93] Menaldino D. S. Bushnev A. Sun A. Liotta D. C. Symolon H. Desai K. Dillehay D. L. Peng Q. Wang E. Allegood J. Trotman-Pruett S. Sullards M. C. Merrill A. H. Jr. (2003) Sphingoid bases and *de novo* ceramide synthesis: enzymes involved, pharmacology and mechanisms of action. Pharmacol. Res. 47, 373–381 12676511 10.1016/s1043-6618(03)00054-9

[94] Vesper H. Schmelz E. M. Nikolova-Karakashian M. N. Dillehay D. L. Lynch D. V. Merrill A. H. Jr. (1999) Sphingolipids in food and the emerging importance of sphingolipids to nutrition. J. Nutr. 129, 1239–1250 10395583 10.1093/jn/129.7.1239

[95] Degagné E. Pandurangan A. Bandhuvula P. Kumar A. Eltanawy A. Zhang M. Yoshinaga Y. Nefedov M. de Jong P. J. Fong L. G. Young S. G. Bittman R. Ahmedi Y. Saba J. D. (2014) Sphingosine-1-phosphate lyase downregulation promotes colon carcinogenesis through STAT3-activated microRNAs. J. Clin. Invest. 124, 5368–5384 25347472 10.1172/JCI74188 PMC4348973

[96] Sugawara T. Kinoshita M. Ohnishi M. Tsuzuki T. Miyazawa T. Nagata J. Hirata T. Saito M. (2004) Efflux of sphingoid bases by P-glycoprotein in human intestinal Caco-2 cells. Biosci. Biotechnol. Biochem. 68, 2541–2546 15618625 10.1271/bbb.68.2541

[97] McQuiston T. J. Haller C. Del Poeta M. (2006) Sphingolipids as targets for microbial infections. Mini Rev. Med. Chem. 6, 671–680 16787378 10.2174/138955706777435634

[98] Thevissen K. Francois I. E. Aerts A. M. Cammue B. P. (2005) Fungal sphingolipids as targets for the development of selective antifungal therapeutics. Curr. Drug Targets 6, 923–928 16375675 10.2174/138945005774912771

[99] Fox T. E. Finnegan C. M. Blumenthal R. Kester M. (2006) The clinical potential of sphingolipid-based therapeutics. Cell. Mol. Life Sci. 63, 1017–1023 16568241 10.1007/s00018-005-5543-z PMC11136019

[100] Zeidan Y. H. Hannun Y. A. (2007) Translational aspects of sphingolipid metabolism. Trends Mol. Med. 13, 327–336 17588815 10.1016/j.molmed.2007.06.002

